# Clinical, hormonal, and biochemical characteristics of 70 chinese children with moderate to severe type 1 diabetic ketoacidosis

**DOI:** 10.1186/s12902-022-01227-9

**Published:** 2022-12-02

**Authors:** Qingxu Liu, Xiaoqin Yin, Pin Li

**Affiliations:** Department of Endocrinology, Shanghai Children’s Hospital, School of medicine, Shanghai Jiao Tong University, 200062 Shanghai, People’s Republic of China

**Keywords:** Diabetic ketoacidosis, Type 1 diabetes mellitus, Thyroid function, Blood lipids, Ionized calcium

## Abstract

**Background:**

Diabetic ketoacidosis (DKA) is one of the most severe acute complications of type 1 diabetes mellitus (T1DM). Patients with DKA of different severities may have different clinical manifestations, serum biochemical levels and hormone changes.

**Methods:**

We retrospectively evaluated the clinical manifestations, serum hormone levels, and biochemical levels of 70 Chinese patients with moderate to severe type 1 DKA in the acute and recovery phases admitted to Shanghai Children’s Hospital from 2015 to 2020.

**Results:**

The time required for acidosis correction in 37 patients with severe DKA was 5.9 h longer than that in 33 patients with moderate DKA (*P* < 0.001). In addition, serum levels of serum ionized calcium (*P* = 0.003), free triiodothyronine (FT3) (*P* = 0.029), white blood cells (WBCs) (*P* = 0.044), and triglycerides (TGs) (*P* = 0.002) were significantly different between patients with moderate and severe DKA. Serum levels of ionized calcium decreased significantly after recovery from severe DKA. Within 1 week, thyroid hormone and blood lipid levels recovered to normal ranges without intervention.

**Conclusion:**

Patients with severe DKA had higher acidosis correction times, higher WBC counts, TGs and ionized calcium levels, and lower FT3 levels than patients with moderate DKA. No additional intervention was required for thyroid hormone, and blood lipid and serum ionized calcium levels recovered to the normal range.

## Background

Diabetic ketoacidosis (DKA) is one of the most severe acute complications of type 1 diabetes mellitus (T1DM), an autoimmune disease characterized by an absolute deficiency of insulin and resultant hyperglycaemia. Several studies have been conducted in adults [1, 2], and these studies showed that leukocyte counts can add valuable information to reflect the presence of hyperglycaemic crisis [2], but there are few comparative studies on children with different severities of type 1 DKA [3]. Therefore, this study aimed to retrospectively assess the clinical manifestations, serum hormone levels, and biochemical levels of children with moderate to severe type 1 DKA during the acute and recovery phases to lay the foundation for a better understanding of DKA.

### Patients and methods

#### Patients

Informed parental consent, patient consent, and approval (ethical approval number: 2020R132-E01) from the Hospital Ethics Committee were obtained before initiating the study. This study recruited 70 children with new-onset T1DM and moderate to severe ketoacidosis from the Department of Endocrinology at Shanghai Children’s Hospital, from 2015 to 2020. All patients met the diagnostic criteria for T1DM with diabetes symptoms, blood glucose ≥ 11.1 mmol/L, and low insulin and C-peptide levels [4]. Participants had no family history of diabetes, goitre, or obesity. Their DKA was diagnosed according to the consensus guidelines proposed by ISPAD [4]. The severity of DKA was divided into mild: venous pH < 7.3 or serum bicarbonate < 15 mmol/L; moderate: pH < 7.2, serum bicarbonate < 10 mmol/L; and severe: pH < 7.1, serum bicarbonate < 5 mmol/L. Because mild DKA can be treated with subcutaneous insulin in the general ward, children with mild DKA were not included in the study. The moderate and severe DKA groups included 33 and 37 patients, respectively.

### Clinical manifestations

We evaluated clinical manifestations in the two groups by comparing differences in sex, age, body mass index (BMI), severity of hyperglycaemia (blood glucose concentration > 33.3 mmol/L) [4], presence of euglycaemic DKA (blood glucose concentration < 11.1 mmol/L) [5], low T3 syndrome, hypercholesterolemia, and hypertriglyceridaemia. C-peptide and insulin analyses were performed randomly before intravenous insulin administration. After initial rehydration, intravenous insulin was administered at a rate of 0.05–0.1 U/kg/h according to the latest guidelines [4]. Insulin and liquid infusion were adjusted according to the degree of dehydration correction, blood gas analysis, and blood glucose levels. Finally, we compared the time for acidosis correction and insulin dosage required to treat DKA after acidosis correction.

### Hormonal and biochemical analyses

Blood biochemistry and blood gas analyses were conducted, and the following parameters were recorded for all patients: blood white blood cell (WBC) count and levels of insulin, C-peptide, blood glucose, glycosylated haemoglobin (HbA1c), diabetes autoantibodies, electrolytes, blood calcium, vitamin D, free triiodothyronine (FT3) with the normal range from 3.58 to 6.92 pmol/L, free thyroxine (FT4) with the normal range from 9.6 to 14.5 pmol/L, triiodothyronine (TT3) with the normal range from 1.34 to 3.7 nmol/L, thyroxine (TT4) with the normal range from 64.3 to 158.7 nmol/L, thyroid-stimulating hormone (TSH) with the normal range from 0.9 to 4.0 µIU/ml, triglycerides (TGs) with the normal range from 0 to 1.7 mmol/L, total cholesterol (TC) with the normal range from 0 to 5.72 mmol/L, high-density lipoprotein (HDL) with the normal range from 0.9 to 1.55 mmol/L, and low-density lipoprotein (LDL) levels with the normal range from 0 to 3.12 mmol/L. C-peptide and insulin analyses were performed randomly before intravenous insulin administration. Electrolytes, blood calcium, blood glucose, TGs, TC, HDL, and LDL were evaluated using detection kits (Beckman Coulter, Brea, CA, USA) and measured with an automatic biochemical analyser (AU5800 analyser, Beckman Coulter, Brea, CA, USA). Thyroid hormone concentrations were evaluated using detection kits (Beckman Coulter, Brea, CA, USA) and measured using an automatic immunoluminescence analyser (Unicel DxI 800, Beckman Coulter, Brea, CA, USA). C-peptide, insulin, and vitamin D levels were evaluated using detection kits (Roche Diagnostics, Mannheim, Germany) and measured using an automatic electrochemiluminescence immunoassay analyser (Cobas e601 analyzer, Roche Diagnostics, Mannheim, Germany). HbA1c levels were evaluated using detection kits (Tosoh Co., Tokyo, Japan) and measured using an automatic HbA1c analyser (HLC-723GX analyser, Tosoh Co., Tokyo, Japan). Diabetes autoantibodies were evaluated using detection kits (Yhlo Biotech Co., Ltd., Shenzhen, China) and measured using an automatic immunoluminescence analyser (iFlash 3000, Yhlo Biotech Co., Ltd., Shenzhen, China).

### Statistical analysis

SPSS 26.0 software, manufactured by International Business Machines Corporation, was used to analyse the data. Nonparametric data were analysed using the Mann–Whitney U test and are presented as the median (25th and 75th percentiles). Normally distributed data were analysed using the t test and are presented as the mean ± standard deviation. Spearman’s rank correlation coefficient was used for correlation regression analysis, and Fisher’s test was used to compare ratios. A *P value* < 0.05 was considered to indicate a significant difference, with significance indicated as follows: *P < 0.05 and **P < 0.01.

## Results

### Clinical manifestations

In this study, 37 (52.86%) patients presented with severe DKA, 33 (47.14%) presented with moderate DKA, and 45 (64.29%) and 25 (35.71%) patients were girls and boys, respectively. All patients had polydipsia, polyuria, and dehydration, while only some of them had nausea, abdominal pain, and Kussmaul breathing. The mean time for acidosis correction in patients with severe DKA was 15.41 ± 0.83 h, which was 5.9 h longer than that in patients with moderate DKA (9.50 ± 0.76 h, *P* < 0.001). After acidosis correction, the daily subcutaneous insulin dosage for DKA patients was close to 1 U/kg/d in both groups. The mean age of patients with severe DKA was 6.90 years of age while that of patients in the moderate DKA group was 7.95 years of age. Meanwhile, the BMI of patients with severe DKA was 15.31 kg/m^2^ and that of patients with moderate DKA was 15.57 kg/m^2^. However, sex, age, BMI, insulin dosage, severity of hyperglycaemia, and presence of euglycaemic DKA, low T3 syndrome, hypercholesterolemia, and hypertriglyceridaemia showed no significant differences between the two groups (Table 1).

**Table 1:** Differences in clinical manifestations of DKA with different severities

Clinical manifestations	Severe DKA	Moderate DKA	*P* value
Boy	16 (43.24%)	9 (27.27%)	0.214
Girl	21 (56.76%)	24 (72.73%)	
Glucose level ≤ 33.3 mmol/L	31 (83.78%)	31 (93.94%)	0.266
Glucose level > 33.3 mmol/L	6 (16.22%)	2 (6.0%)	
Euglycaemic DKA	0 (0)	1 (3.0%)	0.471
Low T3 syndrome	36 (97.30%)	31 (93.4%)	0.598
Hypercholesterolemia	15 (40.54%)	9 (27.27%)	0.315
Hypertriglyceridaemia	27 (72.97%)	18 (54.55%)	0.137
Elevated diabetes autoantibodies	13 (35.14%)	11 (33.33%)	> 0.999

### Hormonal and biochemical levels

**Fig. 1:**
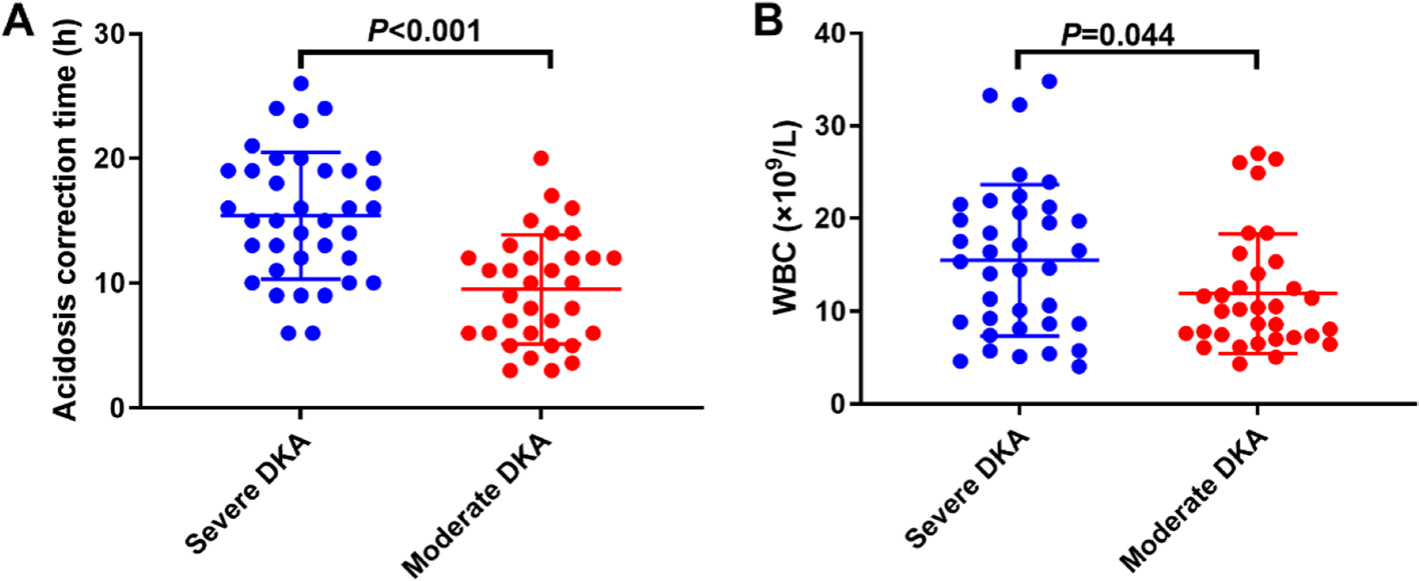
Differences in acidosis correction time and WBC count between severe and moderate DKA patients

The severe DKA group had significantly higher WBC count (15.5 ± 1.34 × 10^9^/L) than the moderate DKA group (11.87 ± 1.12 × 10^9^/L, *P* = 0.044), as well as significantly lower pH (*P* < 0.001), bicarbonate ion (HCO3^−^), and carbon dioxide partial pressure (PaCO2) levels. The hormonal levels of C-peptide, insulin and vitamin D and the biochemical levels of serum sodium, serum potassium, total calcium, HbA1c, blood glucose and blood lactate exhibited no significant difference between the two groups (Table 2; Fig. 1).

**Table 2:** Clinical manifestations and hormonal and biochemical levels of patients with moderate and severe DKA

Indices	Severe DKA (*n* = 37)	Moderate DKA(*n* = 33)	*P* value
Age (years)	6.90 ± 0.61	7.95 ± 0.64	0.243
Acidosis correction time (h)	15.41 ± 0.83	9.50 ± 0.76	< 0.001
Insulin dosage (U/kg/d)	0.99 ± 0.04	1.01 ± 0.03	0.754
BMI (kg/m^2^)	15.31 (14.18–16.54)	15.57 (13.33–17.63)	0.890
Blood pH	6.99 ± 0.02	7.19 ± 0.01	< 0.001
HCO3^−^ (mmol/L)	3.5 (3–4.25)	7.8 (6.1–9.4)	< 0.001
Lac (mmol/L)	1.6 (1.25–1.95)	1.4(0.95–2.6)	0.430
Glu (mmol/L)	26.06 ± 1.11	23.89 ± 1.06	0.166
Sodium (mmol/L)	133.2 ± 0.93	132.5 ± 0.74	0.595
Potassium (mmol/L)	4.24 ± 0.16	4.39 ± 0.13	0.493
Total calcium (mmol/L)	2.21 (2.09–2.45)	2.26 (2.1–2.34)	0.932
WBC (×10^9^/L)	15.5 ± 1.34	11.87 ± 1.12	0.044
Vitamin D (nmol/L)	31.02 ± 5.49	35.37 ± 6.52	0.617
HbA1c (%)	13 (11.35–14.8)	12.8 (11.25–13.85)	0.816
Insulin (pmol/L)	7.54 ± 0.61	6.86 ± 0.91	0.533
C-peptide (ng/mL)	0.08 (0.05–0.13)	0.07 (0.03–0.15)	0.666

**Fig. 2:**
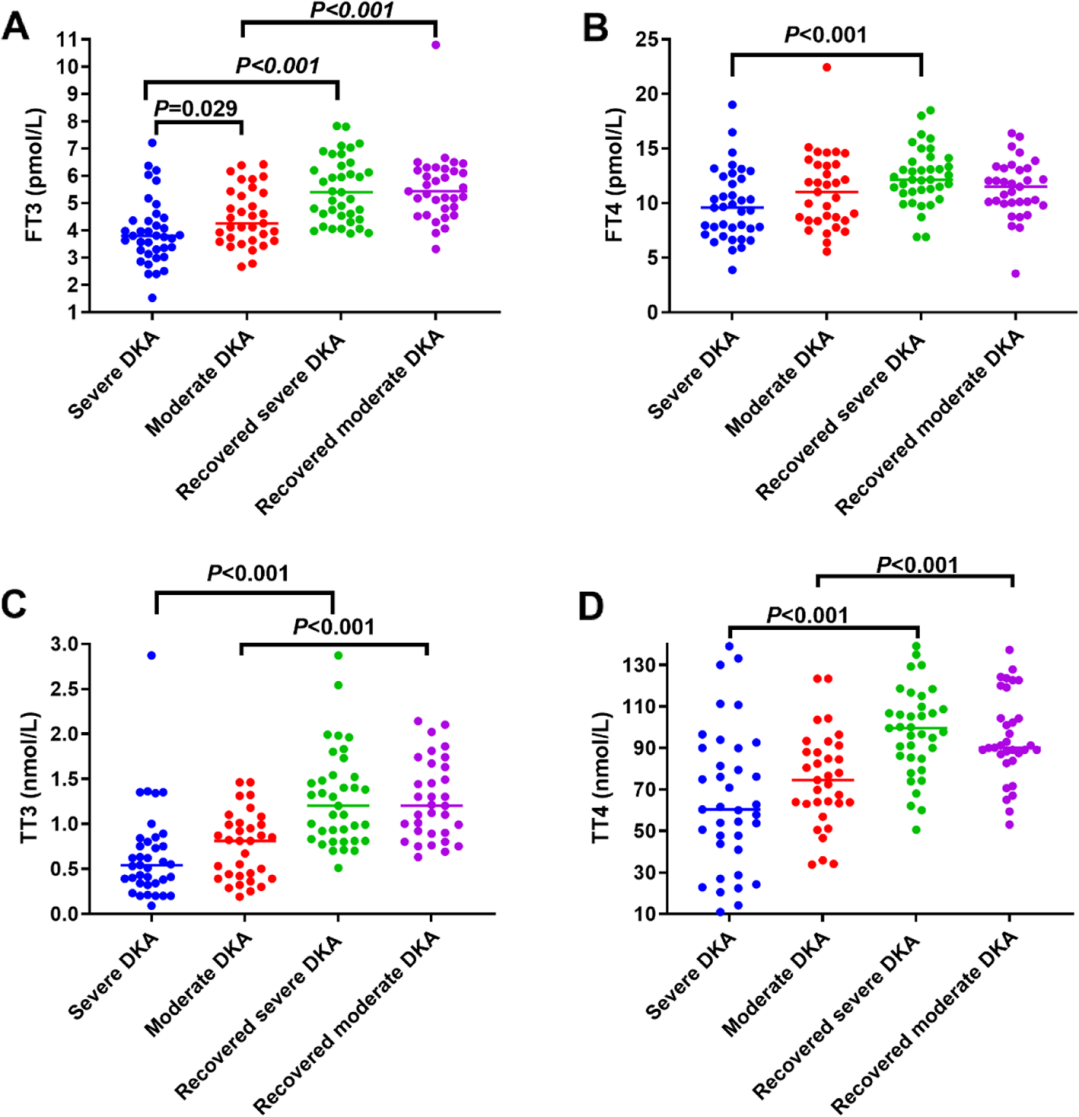
Differences in thyroid hormone levels in acute and recovered DKA stages

**Fig. 3:**
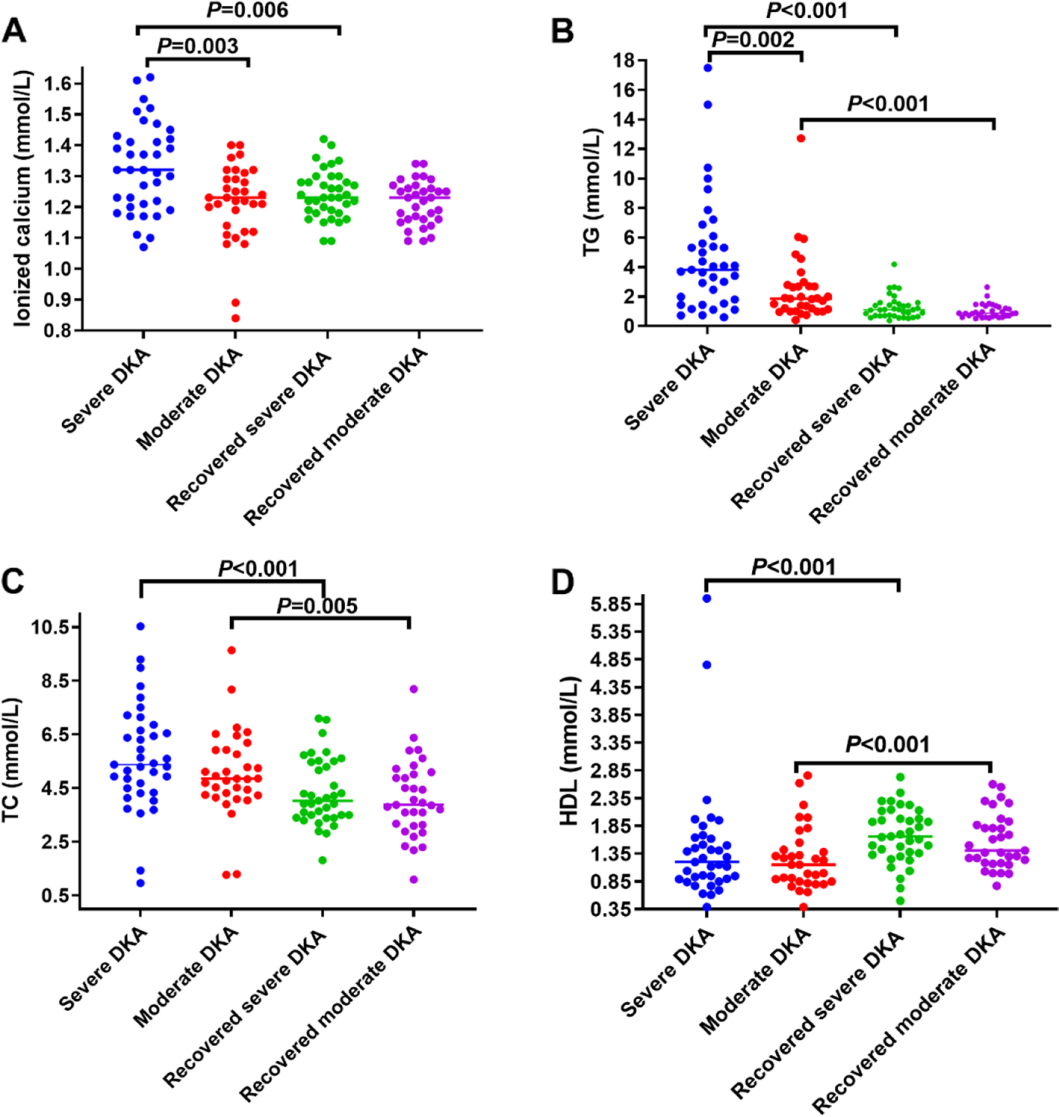
Differences in biochemical levels in acute and recovered DKA stages

All thyroid hormone levels in the severe DKA group were lower than those in the moderate DKA group, as were FT3 levels (*P* = 0.029); however, FT4, TT3, TT4, and TSH levels showed no significant differences between the groups. After acidosis correction, FT3, TT3, and TT4 levels in the two groups were significantly increased within 1 week compared with the levels in the acute phase (*P* < 0.001). FT4 levels in patients with severe DKA increased significantly during the recovery phase (*P* < 0.001), while FT4 levels in patients with moderate DKA showed no significant differences during the recovery phase. In addition, there were no significant differences in the levels of the five thyroid hormones between the two groups during the recovery phase (Table 3; Fig. 2). The level of serum ionized calcium in the severe DKA group was higher than that in the moderate DKA group (*P* = 0.003). After acidosis correction, the level of serum ionized calcium decreased significantly in the severe DKA group (*P* = 0.006). Regarding blood lipids, the level of TGs in the severe DKA group was higher than that in the moderate DKA group (*P* = 0.002). After acidosis correction, TG, TC, HDL, and LDL levels returned to normal ranges within a week. TG (*P* < 0.001) and TC (*P* < 0.001) levels in severe DKA patients and TG (*P* < 0.001) and TC (*P* = 0.005) levels in moderate DKA patients decreased significantly compared with those in the acute phase, while HDL levels increased significantly in both groups (*P* < 0.001). LDL levels were decreased but showed no significant differences compared with the levels in the acute phase. In addition, there were no significant differences in the levels of TGs, TC, LDL, or HDL between the two groups during the recovery phase. In addition, the levels of serum ionized calcium in some patients in the two groups were elevated in the acute phase of DKA, especially in the severe DKA group (*P* = 0.003). After acidosis correction, the serum ionized calcium levels decreased significantly in the severe DKA group (*P* = 0.006), while those in the moderate DKA group did not decrease significantly compared with those in the acute phase (Table 3; Fig. 3).

**Table 3:** Hormonal and biochemical levels in acute and recovered DKA patients

Indices	Severe DKA	Moderate DKA	*P* value	Recovered severe DKA	*P* value	Recovered moderate DKA	*P* value
FT3 (pmol/L)	3.8 (3.21–4.41)	4.26 (3.63–5.41)	0.029	5.4 (4.47–6.43)	< 0.001	5.44 (4.68–6.23)	< 0.001
FT4 (pmol/L)	9.59 (7.40–12.34)	11.04 (8.41–13.54)	0.093	12.16 (10.98–13.97)	< 0.001	11.52 (10–13.2)	0.461
TT3 (nmol/L)	0.54 (0.34–0.82)	0.81 (0.40–1.0)	0.106	1.2 (0.82–1.53)	< 0.001	1.2 (0.89–1.65)	< 0.001
TT4 (nmol/L)	60.3 (42.39–89.96)	74.55 (63.2–89.64)	0.365	99.54 (85.06–112.4)	< 0.001	90.11 (85.45–111.7)	< 0.001
TSH (µIU/ml)	1.69 (0.27–3.01)	2.13 (0.73–3.26)	0.863	2.1 (1.25–3.2)	0.320	2.26 (1.44–3.43)	0.131
Ionized calcium (mmol/L)	1.32 (1.20–1.42)	1.23 (1.13–1.3)	0.003	1.23 (1.18–1.29)	0.006	1.23 (1.16–1.26)	0.657
TGs (mmol/L)	3.82 (1.67–5.85)	1.86 (1.08–2.75)	0.002	1.12 (0.70–1.55)	< 0.001	0.87 (0.67–1.33)	< 0.001
TC (mmol/L)	5.38 (4.42–6.76)	4.86 (4.24–5.92)	0.116	4.04 (3.45–5.49)	< 0.001	3.88 (3.12–5.06)	0.005
HDL (mmol/L)	1.2 (0.89–1.63)	1.15 (0.85–1.48)	0.658	1.66 (1.34–2.05)	< 0.001	1.41 (1.18–1.9)	< 0.001
LDL (mmol/L)	2.79 (2.08–3.62)	2.71 (2.31–3.78)	0.719	2.3 (1.95–3.12)	0.153	2.46 (1.9–2.84)	0.067

### Correlation regression analysis of DKA

**Fig. 4:**
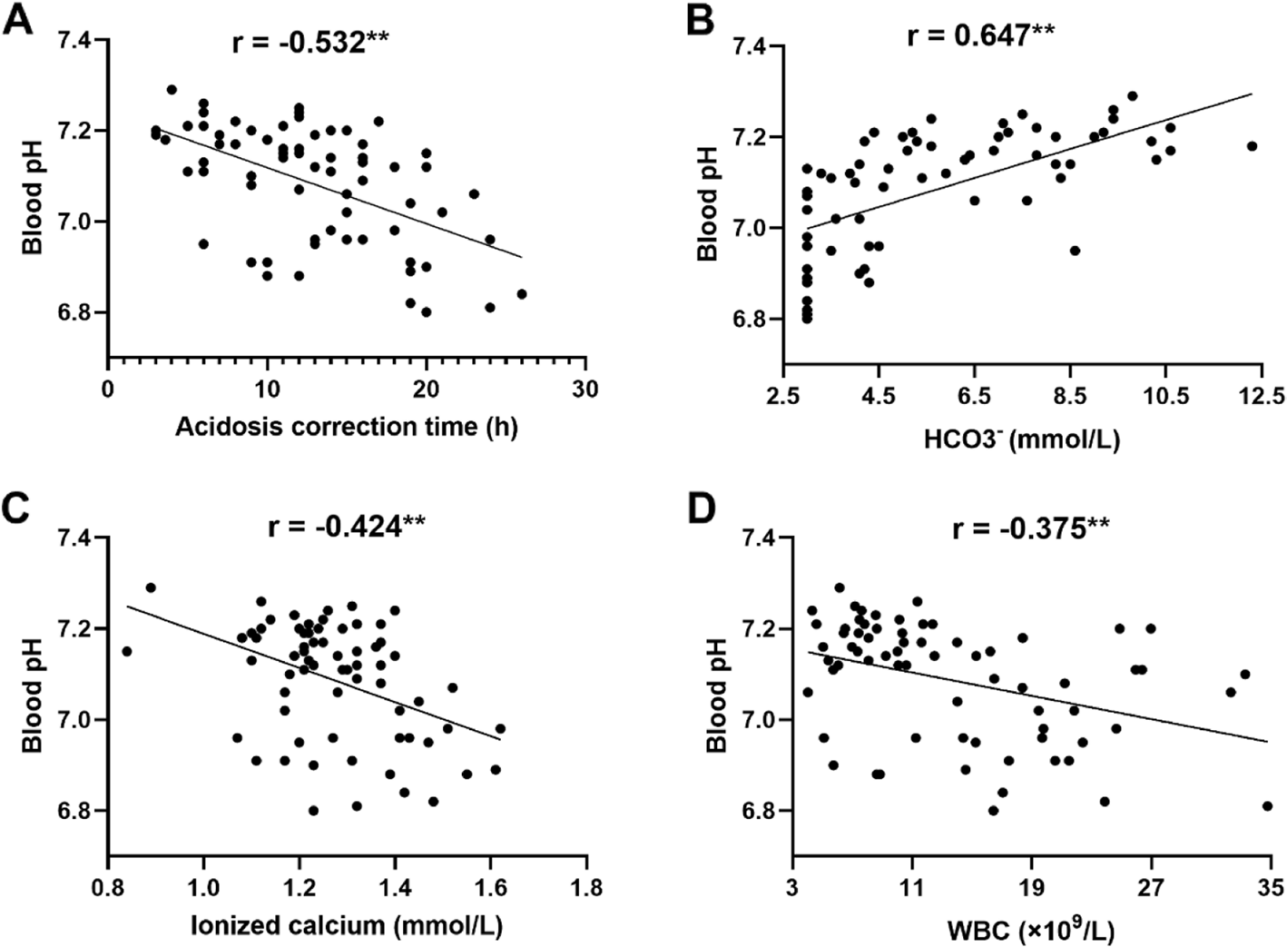
Correlation regression analysis between blood pH and other indices

The time for acidosis correction in patients with severe DKA was negatively correlated with pH (*r* = -0.532, *P* < 0.01) and HCO3^−^ (*r* = -0.248, *P* < 0.01) and positively correlated with ionized calcium (*r* = 0.335, *P* < 0.01) and TG levels (*r* = 0.346, *P* < 0.01) during the acute phase. Throughout the study, pH was positively correlated with HCO3^−^ (*r* = 0.647, *P* < 0.01) and negatively correlated with WBC count (*r* = -0.375, *P* < 0.01) and ionized calcium levels (*r* = -0.424, *P* < 0.01). FT3 was positively correlated with WBC count (*r* = 0.353, *P* < 0.01) and HCO3^−^ (*r* = 0.262, *P* < 0.01), whereas HCO3^−^ was positively correlated with PaCO2 (*r* = 0.863, *P* < 0.01) and negatively correlated with ionized calcium (*r* = -0.458, *P* < 0.01) (Fig. 4)

## Discussion

Epidemiological studies from many parts of the world have shown that the incidence of T1DM is increasing by approximately 2–5% annually [6]. DKA occurs mostly in patients with T1DM and has a peak incidence in individuals aged between 10 and 14 years; however, its clinical manifestations can appear at almost any age [6, 7]. The mean age of children with type 1 diabetic DKA was lower in our study, especially in the severe DKA group. The mean BMI of the two groups was very low, indicating significant dehydration in the patients. Risk factors for DKA include race, younger age, and male sex [8, 9]. There were more female patients than male patients in our study; however, the incidence of severe DKA showed no significant differences with respect to sex. The mean time for acidosis correction in patients with severe DKA was 5.9 h longer than that in patients with moderate DKA, which was expected due to the severity of the disease. DKA may overlap with hyperosmolar hyperglycaemic syndrome (HHS), which is characterized by severe hyperglycaemia and hyperosmolarity and accompanied by circulatory capacity consumption without ketosis [10, 11]. Only one patient developed eugglycaemic DKA in our study, and blood glucose levels in this patient were lower than expected, representing a severe form of DKA [12]. Studies have shown that 3–8.7% of adults with DKA have normal or only slightly elevated blood glucose levels [13]. Euglycaemic DKA may be caused by starvation or a low-carbohydrate high-fat diet [14].

Patients with DKA were in a serious disease state, as they had significantly increased WBC count without evidence of infection. Studies have shown that an elevated WBC count is associated with DKA severity [2] and is likely to be a leukaemoid reaction rather than a systemic inflammatory response. Imbalances in hormone, cytokine, and mediator levels promote an increase in WBC count in response to environmental changes [15]. Few studies have reported vitamin D levels in patients with DKA ; in our study, we found that vitamin D levels in patients with DKA were reduced but did not reach the level of deficiency [16]. Vitamin D signalling is essential for biological processes regulating the immune response [17], and vitamin D deficiency increases the risk of autoimmune diseases [18]. C-peptide levels and β-cell function are better protected in patients who take vitamin D supplements [19]. However, according to our study, vitamin D levels could not distinguish the severity of ketoacidosis. Osmotic diuresis leads to renal potassium and sodium consumption and subsequent systemic consumption. Total calcium levels would decrease in DKA patients because metabolic acidosis itself leads to a negative balance of total calcium [20], and polyuria contributes to calcium loss. In addition, metabolic acidosis contributes to the increase in serum ionized calcium levels.

Regarding thyroid hormones, many patients in our study showed obvious thyroid dysfunction, especially through the development of low T3 syndrome, which is a type of transient thyroid dysfunction characterized by low T3 levels, also known as euthyroid sick syndrome (ESS). When the body is impacted by serious diseases, it reduces thyroidal hormone synthesis to reduce the metabolic rate, and consequently, T3 is transformed into rT3. Children with T1DM and ESS are more prone to DKA, poor metabolic control, and poor glycaemic control than the general population [21, 22]. Previous studies have also shown that serum T4 and T3 levels in patients with metabolic acidosis are decreased; the lower the pH is, the lower the level of FT3 [23]. FT3 and FT4 levels were positively correlated with HCO3^−^ in our study, which is consistent with the results of previous studies [24, 25]. We compared these values with the normal range of thyroid hormones. TT3 and FT3 were affected, and many patients (67/70) suffered from low T3 syndrome, which was consistent with findings of a previous study [23]. Therefore, acidosis severity was the main reason for the decrease in FT3 levels. With insulin treatment, the degree of recovery between the two groups was approximately the same at 1 week. Hormone levels in most patients returned to normal quickly without thyroxine supplementation, which indicated that ESS did not require additional treatment. When the primary disease is cured or exhibits significant remission, ESS resolves itself.

Compared with the insulin levels required to affect blood glucose levels, the insulin levels required for adipolysis are lower [26]. We demonstrated that hyperlipidaemia was common in DKA patients and occurred primarily through hypertriglyceridaemia and hypercholesterolemia. In addition, the severity of acidosis was the main reason for hypertriglyceridaemia, which might be because most of the body fat was stored in the form of TGs. After 1 week of intensive insulin treatment, blood lipid levels in the two groups decreased significantly to the normal range, which was mainly manifested by the decrease in TG and cholesterol levels. Therefore, similar to patients with ESS, those with hyperlipidaemia recovered spontaneously without treatment.

In this study, we found that the time required for acidosis correction in patients with severe DKA was 5.9 h longer than that in patients with moderate DKA. In addition, patients with severe DKA had lower FT3 levels and higher WBC counts, TGs and ionized calcium levels than those with moderate DKA. No additional intervention was required for thyroid hormones, and blood lipids and serum ionized calcium recovered to the normal range.

## Conclusion

We investigated the clinical manifestations, serum hormone levels, and biochemical levels of 70 Chinese children with type 1 DKA of different severities. Patients with severe DKA had a longer time to achieve acidosis correction, lower FT3 levels, and higher WBC counts, TGs and ionized calcium levels than patients with moderate DKA. Serum ionized calcium levels decreased significantly after acidosis correction in patients with severe DKA. During the recovery stage, thyroid hormone and blood lipid levels rapidly recovered to normal ranges. These findings provide deeper insight into DKA and contribute to its clinical assessment and treatment.

## Data Availability

The datasets used and analysed during the current study are available from the corresponding author upon reasonable request.
